# Immunological and molecular signatures of carbamazepine-induced maculopapular exanthema

**DOI:** 10.3389/fimmu.2026.1818888

**Published:** 2026-06-26

**Authors:** Kerry A. Mullan, Stephanie Davies, Kimberley Teoh, Hayley L. Tucker, Jeffrey W. Lai, Alison Anderson, Marian Todaro, Ching-Ching Ng, Kheng-Seang Lim, Johannes S. Kern, Patrick Kwan, Patricia T. Illing, Anthony W. Purcell, Nicole A. Mifsud

**Affiliations:** 1 Department of Biochemistry and Molecular Biology, Infection and Immunity Programs, Biomedicine Discovery Institute, Monash University, Clayton, VIC, Australia; 2 Department of Electrical and Electronic Engineering, University of Melbourne, Melbourne, VIC, Australia; 3 Faculty of Medicine, The University of Melbourne, Melbourne, VIC, Australia; 4 Department of Neuroscience, School of Translational Medicine, Monash University, Melbourne, VIC, Australia; 5 Institute of Biological Sciences, Faculty of Science, Universiti Malaya, Kuala Lumpur, Malaysia; 6 Division of Neurology, Department of Medicine, Faculty of Medicine, Universiti Malaya, Kuala Lumpur, Malaysia; 7 Department of Dermatology, Bayside Health, The Alfred and Department of Immunology, The School of Translational Medicine, Monash University, Melbourne, VIC, Australia; 8 Department of Neurology, Alfred Health, Melbourne, VIC, Australia; 9 Department of Medicine and Therapeutics, Chinese University of Hong Kong, Prince of Wales Hospital, Hong Kong, China

**Keywords:** carbamazepine, drug hypersensitivity reaction, maculopapular exanthema, Stevens–Johnson syndrome, T-cell receptor, T-cells, transcriptomics

## Abstract

Cutaneous adverse drug reactions to carbamazepine range from mild maculopapular exanthema to life-threatening syndromes such as Stevens–Johnson syndrome/toxic epidermal necrolysis. While the immunological basis for HLA-B*15:02-restricted carbamazepine-induced SJS/TEN has received greater attention, mechanisms underlying mild maculopapular exanthema remain poorly understood. In this study, we examined circulating T-cell immune recognition and transcriptomic signatures in the blood of patients with carbamazepine-induced maculopapular exanthema during active and resolved disease and compared these profiles with severe reactions. Peripheral blood was collected from patients with active (< 9 days post-diagnosis; *n* = 5) or resolved (> 2 months post-resolution; *n* = 10) carbamazepine-induced maculopapular exanthema, carbamazepine-tolerant controls (*n* = 11), and resolved carbamazepine-induced Stevens–Johnson syndrome cases (*n* = 11). T-cell reactivity, T-cell receptor clonotypes, and global transcriptional profiles were assessed using cytokine assays, sequencing, and bulk RNA transcriptomics. Both active and resolved maculopapular exanthema patients exhibited polyclonal CD8^+^ T-cell activation. Resolved cases displayed recognition of related tricyclic compounds and private CD8^+^ T-cell receptor clonotypes. Transcriptomics revealed enrichment of complement, interferon, and CD4^+^ T-cell-associated chemokine pathways in active disease, with persistent TNF and NF-κB signalling in resolved cases. Both disease states showed reduced IGHA2 expression, indicating IgA^+^ B-cell dysregulation. Compared with severe hypersensitivity reactions, maculopapular exanthema lacked sustained proinflammatory signalling, highlighting divergent long-term immune activation. Together, we demonstrated that carbamazepine-induced maculopapular exanthema is driven by polyclonal CD8^+^ T-cell responses in conjunction with innate immune activation and exhibits distinct molecular signatures from severe reactions, providing mechanistic insight into the milder clinical phenotype.

## 1 Introduction

Cutaneous adverse drug reactions (cADRs) span from a skin rash known as maculopapular exanthema (MPE) to severe multisystem syndromes involving skin and mucosal detachment, as in the case of Stevens–Johnson syndrome (SJS) and toxic epidermal necrolysis (TEN), as well as drug reaction with eosinophilia and systemic symptoms (DRESS) (1). A leading class of prescription drugs implicated in cADRs is antiseizure medications (ASMs), which include carbamazepine (CBZ), lamotrigine (LTG), and phenytoin (PHT) (2). CBZ is an effective first-line treatment option for epilepsy, neuropathic pain, and the management of bipolar affective disorder (3). Despite this efficacy, CBZ-induced cADRs occur at a rate of one in 10 for milder clinical manifestations such as MPE (4), with more severe pathologies of DRESS and SJS/TEN estimated at rates of one in 1,000 to one in 10,000 (5, 6), which increases to one in 400 for SJS/TEN in patients of Han Chinese descent (7). Once diagnosed, drug withdrawal resolves symptoms in mild disease, although the more severe reactions require significant clinical intervention.

Both MPE and SJS are categorised as type IV delayed-type drug hypersensitivity reactions (DHRs) and can be distinguished based on immune mediators that promote immunopathology (8). For instance, SJS is type IVc involving CD8^+^ T-cell cytotoxicity mediated by production of granzyme B, perforin, Fas ligand, and granulysin (9–11). While MPE spans type IVa involving CD4^+^ Th1 cells activating macrophages/monocytes via proinflammatory cytokine release (interferon γ [IFN-γ], tumour necrosis factor [TNF]) (12, 13), type IVb involving CD4^+^ Th2 cell activation of eosinophils via production of interleukin (IL)-4, IL-5, IL-13, and eotaxins, as well as type IVc, involving both CD4^+^ Th1 cells and cytotoxic CD8^+^ T cells (14).

A major genetic risk association for the development of both mild and severe CBZ-induced pathologies is specific human leukocyte antigen (HLA) allotypes (15, 16). The highest risk association for SJS/TEN is HLA-B*15:02 carriage in the Han Chinese population at an odds ratio (OR) of 895 (17), which also extends to other Asian ethnicities, including Indian (OR: 71.40) (18), Malaysian (OR: 221.00) (19), and Thai (OR: 70.91) (20). For MPE, HLA-A*31:01 carriage in European and Han Chinese populations has ORs between 8.33 and 17.5 (21, 22). Interestingly, there appears to be a distinct dichotomy for HLA associations, with severe pathologies of SJS/TEN having greater restriction towards allotypes within the HLA-B75 serotype [i.e., B*15:02, B*15:08, B*15:11, B*15:18, B*15:21; reviewed in (23)]. While the milder MPE reaction demonstrates greater promiscuity involving HLA-A*31:01 and HLA-A*24:02 [reviewed in (23)], as well as more recent reports in HLA-B*13:01 [Southern Han Chinese, Thai (24)], HLA-B*38:02 [Southern Han Chinese (25)], HLA-B*58:01 [Thai (20)], or a complete lack of HLA association [Northeast Han Chinese (26)].

Several studies have shown that CBZ activates T cells via pharmacological interaction with immune receptors (p.i.), with the small molecule drug forming non-covalent interactions with either the peptide/HLA (pHLA) complex and/or the T-cell receptor (TCR) at the cell surface (9, 27). This labile interaction does not require antigen processing pathways; therefore, it is not thought to perturb the peptide cargo presented by the HLA molecule (27). There is a plethora of studies cataloguing CBZ-induced T-cell-mediated responses in both mild MPE and the more severe forms of DRESS and SJS/TEN [reviewed in (23)]. However, there is increasing interest in understanding the role of innate immune mediators in the pathogenesis of these cADRs. For instance, evidence of NK cell effector mechanisms promoting keratinocyte cell death involving granulysin has been observed in the blister fluid of SJS/TEN patients (11). Furthermore, the role of the complement cascade as part of innate immunity has been suggested to promote severe SJS/TEN pathology, but not milder MPE reactions (28).

Despite the high rate of patients developing drug-induced MPE, individuals rarely present to the hospital, as they often consult their general practitioner and are advised to immediately cease medication use, thereby resolving the DHR. As a consequence, opportunities to identify risk genes via transcriptomics (29) and immunological biomarkers (28) that define the MPE signature have been limited. Additionally, there have been no studies examining the molecular signatures of active versus resolved disease states in MPE cases or their comparison with more severe pathologies to explain their position on the cADR severity spectrum.

Although skin is the primary disease site in MPE, largely driven by tissue-resident T-cell responses (30, 31), peripheral blood remains the most clinically accessible compartment for immune monitoring and biomarker development. In this study, we examined *in vitro* drug-induced T-cell responses (via Th1 cytokine production) and their cellular transcriptome in CBZ-induced MPE cases both during active (a; < 9 days post-diagnosis) and resolved (*r*; > 2 months after disease resolution) stages of disease, as compared to CBZ-tolerant cases (**Supplementary Figure 1**). In both CBZ-induced active MPE (aMPE) and resolved MPE (rMPE) cases, our data showed circulating CD8^+^ T-cell responsiveness. Further examination of rMPE cases demonstrated CD8^+^ T-cell responses towards other tricyclic aromatic compounds and utilisation of private TCR clonotypes recognising CBZ-stimulated pHLA complexes. Next, we compared the transcriptional profiles of peripheral blood mononuclear cells (PBMCs) from aMPE versus rMPE compared with tolerant cases (**Supplementary Figure 1**). Here, aMPE cases presented with an enrichment of both complement and interferon signalling pathways involved in innate immune responses (i.e., characteristic of a type IVa reaction), as well as activation of CD4^+^ T-cell-mediated chemokine and signalling pathways (i.e., characteristic of a type IVc hypersensitivity). While rMPE cases showed significant upregulation of chemokine, cytokine, TNF, and NF-κB signalling pathways linked with type IVc reactions compared with tolerant cases. Interestingly, a significant loss of *IGHA2* associated with IgA^+^ B cells was also observed in all MPE cases, when compared with tolerant cases. Finally, comparisons between CBZ-induced mild versus severe pathology in resolved cases (i.e., rMPE vs. rSJS) showed that rSJS patients maintained significantly upregulated proinflammatory pathways, suggesting these individuals retain a high-risk activated phenotype many years postreaction.

## 2 Methods

### 2.1 Study cohort

A total of five active CBZ-MPE (aMPE) cases, 10 resolved CBZ-MPE (rMPE) cases, and eight CBZ-tolerant (Tol) cases were recruited to the study from The Royal Melbourne Hospital (Victoria, Australia; Human Research Ethics Committee [HREC] ID 2015.073). MPE was diagnosed based on clinical history and characteristic cutaneous morphology, confirmed by dermatologists, with histopathological evaluation performed only when clinically required and not systematically available across the cohort (32, 33). Additionally, 11 resolved CBZ-induced SJS patients (rSJS) and three Tol cases were recruited from either Hong Kong (Joint Chinese University of Hong Kong–New Territories East Cluster Clinical Research Ethics Committee ID CRE-2006.203) or the University of Malaya Medical Centre (HREC 950.49). As described in our previous studies (34, 35), the diagnosis of SJS was based on the criteria by Roujeau and Stern (36), defined by skin detachment in two or more mucosal sites, and was confirmed by dermatologists. In this study, aMPE cases were defined as up to 9 days post-diagnosis, and rMPE or rSJS cases > 2 months following disease resolution. Blood collection and PBMC isolation were performed in each country, with all experiments conducted in Australia. The study cohort demographic and clinical data are shown in **Supplementary Table 1**. Informed consent was obtained from all study participants under the cited institutional ethics approvals.

### 2.2 PBMC isolation and generation of CBZ-induced T cells

PBMCs were isolated from blood samples using Ficoll (GE Healthcare, Uppsala, Sweden) density gradient centrifugation. Samples were either used fresh or cryopreserved in fetal calf serum (FCS) containing 10% DMSO (Sigma-Aldrich, St. Louis, MO, USA) at − 196°C. Cryopreserved PBMCs were quickly thawed at 37°C and washed twice in Roswell Park Memorial Institute (RPMI) 1640 (Gibco, Thermo Fisher Scientific, Waltham, MA, USA) prior to use in immunoassays. CBZ-induced T cells were *in vitro* expanded following PBMC stimulation with 25 μg/mL of CBZ at a density of 5 × 10^6^ cells per 2 mL of complete medium (RPMI 1640 supplemented with 2 mM MEM nonessential amino acid solution [Gibco], 100 mM HEPES [Gibco], 2 mM l-glutamine [Gibco], penicillin/streptomycin [Gibco], 50 μM 2-mercaptoethanol [Sigma-Aldrich], and 10% heat-inactivated human blood group AB serum [Sigma-Aldrich]). From days 4 to 14, T-cell cultures were supplemented with 50 U/mL of recombinant human IL-2 (Peprotech, Rocky Hill, NJ, USA) and subcultured as required for optimal outgrowth.

### 2.3 Antigen-presenting cells

Autologous B lymphoblastoid cell lines (B-LCLs) were generated from PBMCs derived from both cases and healthy controls. Briefly, 1 mL of Epstein–Barr virus supernatant from the B95.8 cell line (37) was added to 5 × 10^6^ PBMCs in 5 mL RF10 (same constituents as complete medium except 10% FCS), with 150 μL of the EBV supernatant/PBMC mixture then aliquoted into a 96-well plate. Cells were supplemented with 25 μL/well of 3.5 μg/mL cyclosporine A (Sigma-Aldrich) in RF10 to a final concentration of 0.5 μg/mL. Weekly, 80 μL of media was discarded, and 100 μL fresh RF10 containing 0.875 μg/mL cyclosporine A was added to each well. Cells were maintained in RF10 once large EBV-transformed B-LCL clusters were visible. Several cell line transfectants were generated from the HLA class I-reduced C1R B-LCL, which has no detectable cell surface expression of HLA-A*02:01, minimal HLA-B*35:03, and normal levels of HLA-C*04:01 and class II loci cell surface expression (38, 39). All cell lines were cultured in RF10, with maintenance of HLA expression being facilitated by selection antibiotics, either 0.5 mg/mL geneticin G418 (Roche Diagnostics, Mannheim, Germany) or 0.3 mg/mL hygromycin B (Thermo Fisher Scientific), as required, except for antigen-presenting cells (APCs) tagged with green fluorescent protein (GFP). Additionally, International HLA and Immunogenetics Workshop (IHIW 90xx cell line series) B-LCLs (40) were also used as APCs (**Supplementary Table 2**). Surface HLA class I expression of cell lines was confirmed using indirect staining with anti-human HLA primary antibodies (produced in-house from hybridomas; **Supplementary Table 3**), followed by staining with a secondary goat antimouse IgG allophycocyanin (AlloPC; Southern Biotech, Birmingham, AL, USA) or phycoerythrin (PE). A total of 30,000 stained cells were acquired on a Becton Dickinson (BD; San Diego, CA, USA) Fortessa X20 flow cytometer and analysed using FlowJo software (version 10, BD) (**Supplementary Figure 2**).

### 2.4 Quantitation of CBZ-induced T-cell responses

Functionality of CBZ-induced T cells was measured using intracellular cytokine staining as previously described (27). Briefly, day 14 CBZ-induced T cells were restimulated with either Dynabeads^®^ Human T-Activator CD3/CD28 beads (Thermo Fisher Scientific) or autologous B-LCLs + 25 μg/mL CBZ (representing positive controls), 25 μg/mL CBZ, APC alone, or APC + 25 μg/mL CBZ at a 2:1 T-cell-to-APC ratio for a total of 6 h. After 2 h, 10 µg/mL brefeldin A (Sigma-Aldrich) was added. Stimulated cells were then surface labelled with LIVE/DEAD^®^ fixable Aqua stain (Thermo Fisher Scientific), CD4 PE (clone RPA-T4, BD Biosciences, Franklin Lakes, NJ, USA), CD8 PerCP-Cy5.5 (clone SK1, BD Biosciences), fixed in 1% paraformaldehyde (ProSciTech, Kirwan, Queensland, Australia) in phosphate-buffered saline (PBS), and then permeabilised with 0.3% Saponin (Sigma-Aldrich) in PBS containing IFN-γ PE-Cy7 (clone B27, BD Biosciences) and TNF V450 (clone MAb11, BD Biosciences). A total of 50,000 lymphocytes were acquired on a BD Fortessa X20 flow cytometer and analysed using FlowJo software (version 10, BD). All monoclonal antibodies were titrated for optimal staining efficiency prior to use. The representative gating strategy is shown in **Supplementary Figure 3**.

### 2.5 Signatures of CBZ-induced αβTCRs and bioinformatics

A single-cell sort was performed to characterise the αβTCR signature of drug-induced T cells using the IFN-γ Secretion Assay–Detection Kit (AlloPC; Miltenyi Biotec, Bergisch Gladbach, Germany). Cryopreserved day 14 T cells were thawed and rested overnight in Complete Medium. T-cell lines (maximum of 5 × 10^6^ cells) were incubated with either APC alone or APC + 25 μg/mL CBZ (2:1 ratio) in RH5 media (same constituents as complete medium, except 5% heat-inactivated human blood group AB serum) for 4 h at 37 °C, 5% CO_2_. Cells were washed in cold wash buffer (0.5% FCS, 2 mM EDTA, pH 8.0 in PBS), centrifuged (285 ×*g*, 5 min, 4 °C), and supernatant aspirated before addition of IFN-γ catch reagent antibody according to manufacturer’s instructions. Cells were incubated on ice for 5 min and topped up to 10 mL with warm RH5 media, and 25 μg/mL CBZ was added. Cells were incubated for 45 min at 37 °C, 5% CO_2_ with rotation. Cells were washed in cold wash buffer, centrifuged, and supernatant aspirated prior to costaining with IFN-γ AlloPC detection reagent and CD8 FITC (clone HIT8a, BD Biosciences). Cells were incubated on ice for 20 min, washed in cold wash buffer, centrifuged, and resuspended in 300 μL cold wash buffer. Single cells were sorted on a BD Influx flow cytometer (FlowCore, Monash University) directly into 96-well PCR plates (Bio-Rad, Hercules, CA, USA) based on CD8^+^IFN-γ^−^ for CBZ-unresponsive T cells (negative control) and CD8^+^IFNγ^+^ for CBZ-induced T cells. Sorted plates were immediately stored at − 80 °C until required. Analysis of paired *TCRα* and *TCRβ* genes was performed by multiplex nested RT-PCR and sequencing of α and β products as described previously (27, 41). Both external and internal rounds of PCR included 40 TRAV and 27 TRBV forward primers, and TRAC and TRBC reverse primers, as detailed elsewhere (41). Sanger sequencing (.seq) and chromatogram (.ab1) output files were uploaded into the TCR_Explore webtool (42) for TCR repertoire analysis. Briefly, each .seq file was converted into a .fasta file and merged in lots of 50 using TCR_Explore for alignment in IMGT/VQUEST (43) and then imported back into the webtool. Chromatograms (.ab1 file) were also uploaded to TCR_Explore and scored for sequence quality by assigning “pass” or “fail”. Sequences that failed, based on either the V-QUEST parameters or the chromatogram score, were manually checked. If sequences were resolvable, the manually resolved sequences were added to the bottom of the file and assigned a “pass”. Alignment sequences and the scored chromatogram file were then combined. This quality control (QC)-checked file was then uploaded to TCR_Explore for automated pairing of α and β chains and merged for analysis using the webtool “Analysis section”.

### 2.6 Bulk RNA-Seq

Total RNA was isolated from PBMCs according to the manufacturer’s instructions (RNeasy Mini Kit; Qiagen, Düsseldorf, Germany). RNA concentration (> 20 ng/μL) and purity (wavelength ratio: 280/260 and 260/230 between 1.8 and 2.2) were assessed using Nanodrop One (Thermo Fisher Scientific). Each sample underwent QC by BGI NGS Laboratory (Tai Po, Hong Kong), and, if it passed, was subjected to RNA-Seq at a depth of 30 million reads of nonstranded with 100 base-pair (bp) end reads on a BGI-500 instrument. FASTQ output files were aligned to the human genome build GRCh38 using the Monash Bioinformatic Platform RNAsik pipeline (version 1.5.0) (44) to produce a count file and QC metrics. One sample, aMPE-3 PBMC, showed biased GC coverage and low feature count assignment (~ 6,500,000 reads) and was excluded from subsequent analysis. Raw count files were loaded into R Studio (R version 4.1.1) for QC and differential expression analysis. The following R packages were used to filter the data: “tidyr” (version 1.1.4) (45), “dplyr” (version 1.0.7) (46), and “tidyverse” (version 1.3.1) (47), which were used to create a consistent format. The raw count data were formatted into a gene-by-sample matrix using “edgeR” (version 3.34.1) (48). Genes derived from X and Y chromosomes were removed, as well as mitochondrial genes, due to their distinct dosage-related expression patterns requiring separate statistical consideration. Next, genes with an average of < 10 counts across all samples were filtered out, as they had limited expression confidence. The remaining 16,948 PBMC genes were used for the final differential expression analysis. Next, PBMC sample outliers were assessed using principal component analysis (PCA), with the first three PCs representing > 80% of the total variation. The threshold for removal of samples was set at ± 2.5 SD from the mean in at least one of these PCs. This identified a single outlier from CBZ-tolerant samples (Tol-1 PBMC; **Table 1**), which was removed from subsequent analyses. This filtered matrix in edgeR was then normalised using the weighted trimmed mean of *M*-values. Here, a generalised linear model (Genewise Negative Binomial Generalised Linear Models [GLM]) was applied using trended dispersion. From this model, two categories at a time (e.g., rSJS vs. Tol) were compared to produce the differential expression file. Next, the log_2_ fold change (logFC), false discovery rate (FDR), and ID for data visualisation and analysis were extracted using the ggVolcanoR webtool (49). Transcripts were deemed significantly differentially expressed with a FDR < 0.10 (i.e., 10%) and a logFC ± 0.58 (1.5-fold difference). These cut-offs, with less FDR thresholds, have been used in studies of complex traits to allow for greater discovery (50). To explore case clustering of the significantly differentially expressed genes (DEGs), an in-house script, heatmap.R, was created using the “ComplexHeatmap” (version 2.9.4) (51) and “circlize” (version 0.4.13) (52) packages. PC plots were created using the ggplot2 package, along with “magrittr” (version 2.0.1) (53), “gridExtra” (version 2.3) (54), “reshape2” (version 1.4.4) (55), and “ggrepel” (version 0.9.1) (56) to improve formatting. Pathway analyses of significantly DEGs were performed using the g:Profiler web-based tool (57).

**Table 1:** Summary of immune and transcriptional changes across CBZ-induced disease states.

Disease	Key T-cell features	Other immune features	Potential implications
aMPE	Reduced γδ T cells (Vδ2γ9) and monocyte chemoattractant signatures	Low *IGHA2* gene expression (IgA^+^ cells)	Impaired T-cell/γδ T-cell surveillance and increased monocyte/complement activity
		*C1Q* gene activation	B cells expressing low to absent levels of IgA2
rMPE	Similar to aMPE	Low *IGHA2* gene expression (IgA^+^ cells)	Persistent low-grade inflammation and reduced checkpoint signaling
		Reduced *CD24* gene expression	
		Neutrophil-associated *CXCL8* and *IL6* gene upregulation	
rSJS	Increased γδ T cells (Vδ2γ9^+^)	Increased *CD24* gene expression	Persistent cytotoxicity and compensatory immune regulation
		*GZMB*, *PRF1*, and *IL6* gene upregulation	

*a*, active; *γδ*, gamma-delta; *Ig*, immunoglobulin; *IL*, interleukin; *MPE*, maculopapular exanthema; *r*, resolved; *SJS*, Stevens–Johnson syndrome.

### 2.7 Code and data availability

Scripts used for differential expression analyses are available on GitHub (https://github.com/KerryAM-R/DHRs_transcript). The PBMC folder includes the following: (1) reformatting, (2) quality control and applying GLM from edgeR, and (3) differential expression analysis and heatmap generation. Public sharing of the transcriptomic data for the edgeR object after filtering and adjustments, as well as the processed GLM file, is available as an.rds object on Zenodo (https://zenodo.org/records/16938115).

### 2.8 Statistics

All data were reported as mean ± standard error of the mean (SEM), unless stated otherwise. Statistical significance was determined by nonparametric one- or two-way analysis of variance (ANOVA) with *post hoc* Tukey’s multiple comparison test (MCT) with ^*^p < 0.05, ^**^p < 0.01, ^***^p < 0.001, and ^****^p < 0.0001 (Prism 10.0, GraphPad software).

## 3 Results

### 3.1 Active CBZ-MPE patients do not exhibit HLA-I allotype-biased CD8^+^ T-cell responses

A total of four patients experiencing CBZ-induced aMPE were examined to determine whether their drug-induced T-cell responses were restricted to specific HLA-I allotypes (aMPE1–4; **Supplementary Table 1**). PBMCs were *in vitro* expanded for 14 days after an initial stimulation with 25 μg/mL CBZ. Drug-induced T cells were then restimulated for 6 h with no drug (untreated), either CD3/CD28 beads or autologous B-LCLs + 25 μg/mL CBZ as a positive control, 25 μg/mL CBZ alone, or HLA-matched APCs in the absence or presence of 25 μg/mL CBZ (**Supplementary Table 2**). CBZ-induced T cells were immunophenotyped to quantify production of proinflammatory Th1 cytokines, IFN-γ and TNF, from both CD8^+^ and CD4^+^ T cells. Here, C1R parental + 25 μg/mL CBZ (C1R + CBZ) was used as the baseline to account for potential HLA class I (B*35:03, C*04:01) and class II (DRB1*12:01, DQB1*03:01, DPB1*04:01) (38, 39) background responses in HLA-transfected (of interest) C1R cell lines. Statistical significance (*p* < 0.05) was measured against this baseline, even if there was significance for each transfected cell line in the absence or presence of the drug.

**Figure 1:**
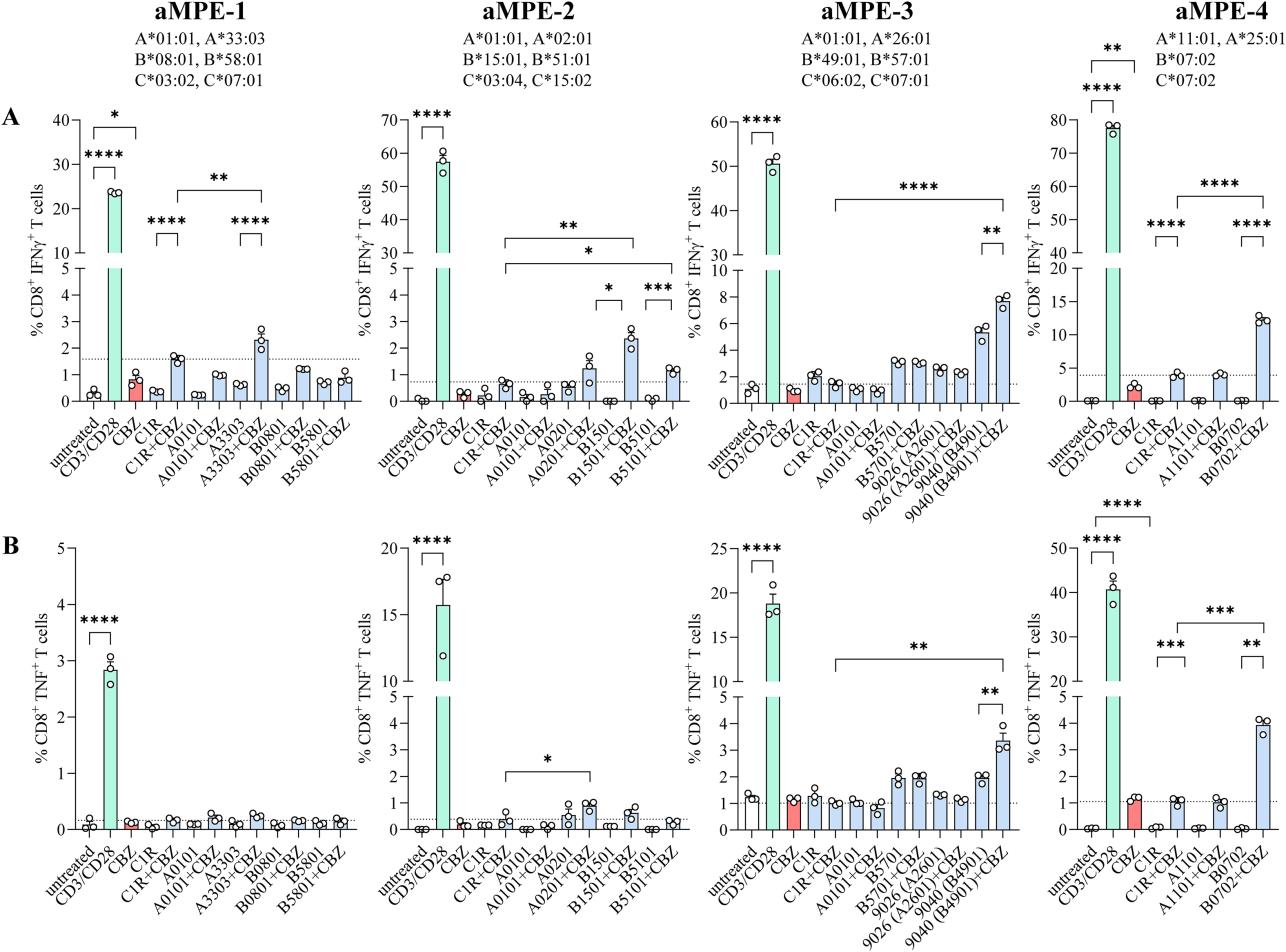
CBZ-induced CD8^+^ T-cell responses in active MPE patients. PBMCs were treated *in vitro* with 25 μg/mL CBZ for 14 days. Drug-induced activation of CD8^+^ T-cells, via production of IFN-γ **(A)** and/or TNF **(B)**, was quantified following a 6-h restimulation with either no drug (untreated), CD3/CD28 beads (non-specific stimulator), 25 μg/mL CBZ, or HLA-matched APCs in the absence or presence of 25 μg/mL CBZ. Triplicate data were acquired on a BD Fortessa X20 flow cytometer and analysed using FlowJo software (version 10, BD). Data are expressed as mean (%) ± standard error of mean (SEM). The dotted line represents the mean value for CD8^+^ T-cell responses to C1R + CBZ treatment. Statistical significance was determined using one-way ANOVA with Tukey’s multiple comparisons test (MCT) for *post hoc* analysis (^*^p < 0.05; ^**^p < 0.01; ^***^p < 0.001; ^****^p < 0.0001; Prism 10.0, GraphPad software). HLA class I genotyping is shown for each case (**Supplementary Table 1**).

Our primary aim was to determine whether CBZ-induced aMPE cases exhibited HLA class I-restricted immune responses, as reported in severe CBZ reactions. Here, we observed enhanced CD8^+^ IFN-γ^+^ T-cell responses primarily towards HLA-B allotypes for aMPE-2 (HLA-B*15:01 and HLA-B*51:01), aMPE-3 (HLA-B*49:01), and aMPE-4 (HLA-B*07:02), with aMPE-1 focused towards HLA-A*33:03 (**Figure 1A**). Mirrored responses, albeit at lower levels, were observed for CD8^+^ TNF^+^ T cells for aMPE-3 and aMPE-4 (**Figure 1B**). However, no significant TNF production was observed for aMPE-1 and aMPE-2, with the latter showing significance towards HLA-A*02:01 but not HLA-B allotypes, likely due to low TNF production. Finally, CD8^+^ T-cell reactivity towards the drug alone was observed in two active patients (aMPE-1, aMPE-4), indicating T–T stimulation by CBZ (**Figures 1A, B**).

**Figure 2:**
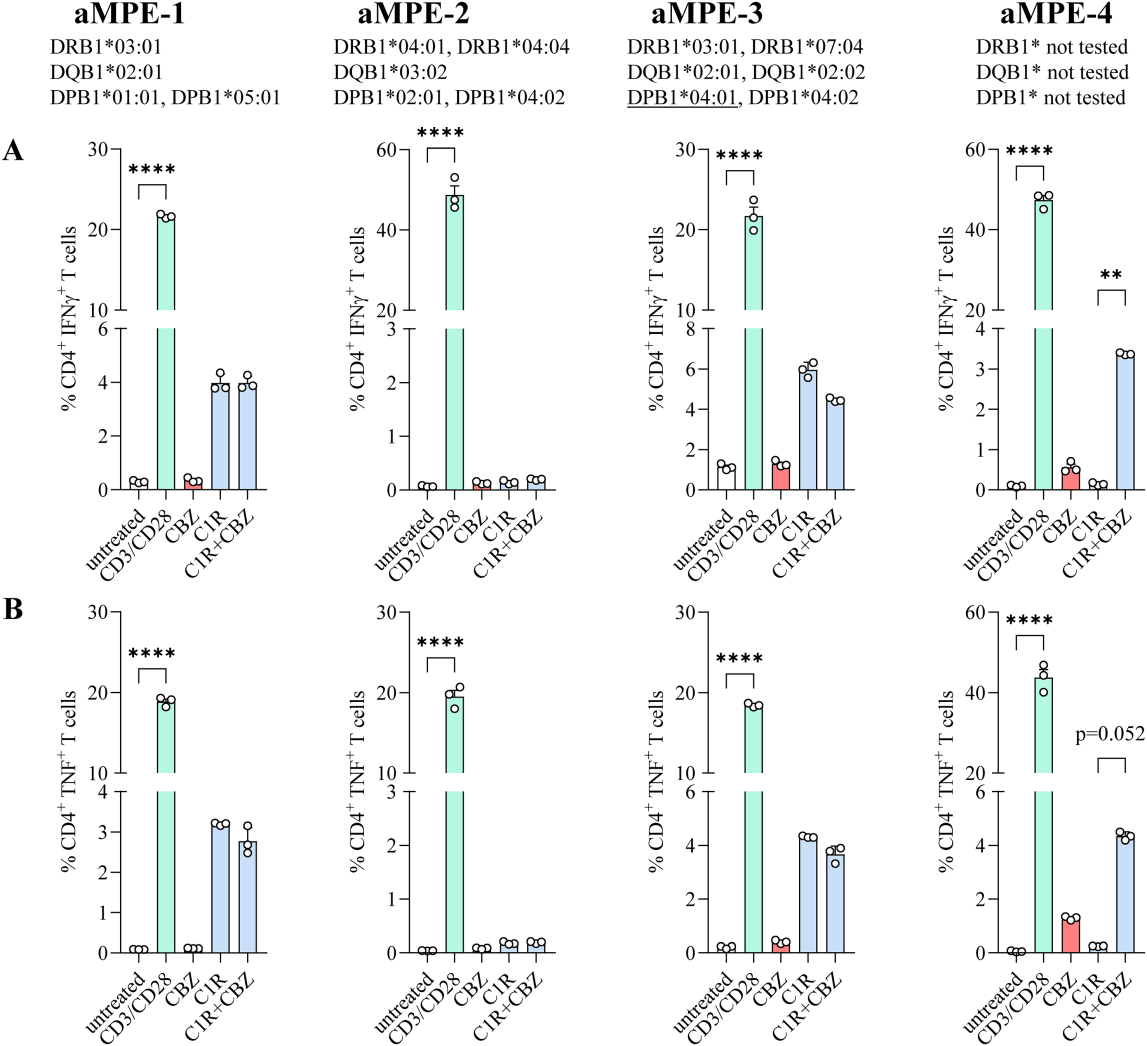
CBZ-induced CD4^+^ T-cell responses in active MPE patients. PBMCs were treated *in vitro* with 25 μg/mL CBZ for 14 days. Drug-induced activation of CD4^+^ T-cells, via production of IFN-γ **(A)** and/or TNF **(B)**, was quantified following a 6-h restimulation with either no drug (untreated), CD3/CD28 beads (non-specific stimulator), 25 μg/mL CBZ, or C1R parental cells in the absence or presence of 25 μg/mL CBZ. Triplicate data were acquired on a BD Fortessa X20 flow cytometer and analysed using FlowJo software (version 10, BD). Data are expressed as mean (%) ± standard error of mean (SEM). The dotted line represents the mean value for CD4^+^ T-cell responses to C1R + CBZ treatment. Statistical significance was determined using one-way ANOVA with Tukey’s multiple comparisons test (MCT) for *post hoc* analysis (^**^p < 0.01; ^****^p < 0.0001; Prism 10.0, GraphPad software). HLA class II genotyping is shown for each case (**Supplementary Table 1**), with underlined allotypes shared with C1R parental cells.

We also evaluated CD4^+^ T-cell responses in this cohort. Although HLA class II-matched APCs were unavailable, CD4^+^ T-cell reactivity to the drug alone or to parental C1R cells in the presence of the drug was assessed. Among these cases, only aMPE-4 showed a significant response, evidenced by IFN-γ production and a near-significant TNF response (*p* = 0.052) (**Figures 2A, B**). Together, these findings suggest that CBZ-induced aMPE in blood was dominated by HLA-B-focused CD8^+^ T-cell responses, with limited CD4^+^ T-cell involvement.

### 3.2 Multiple HLA-I allotypes trigger recall responses in resolved CBZ-MPE patients

**Figure 3:**
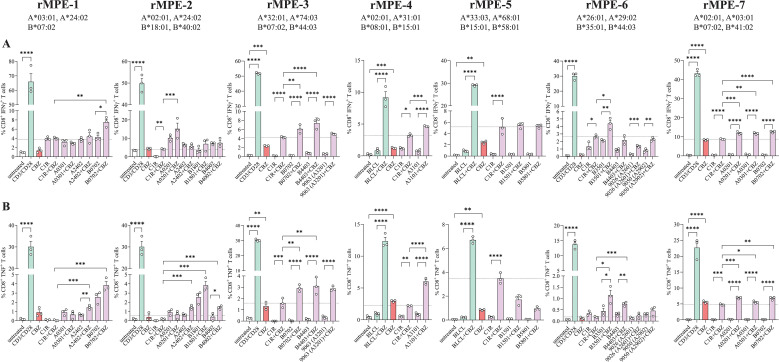
Drug-induced CD8^+^ T-cell recall responses in resolved MPE patients. PBMCs were treated *in vitro* with 25 μg/mL CBZ for 14 days. Drug-induced activation of CD8^+^ T-cells, via production of IFN-γ **(A)** and/or TNF **(B)**, was quantified following a 6-h restimulation with either no drug (untreated), CD3/CD28 beads, or autologous B-LCL + 25 μg/mL CBZ (positive controls), 25 μg/mL CBZ, and HLA-matched APCs in the absence or presence of 25 μg/mL CBZ. Triplicate data were acquired on a BD Fortessa X20 flow cytometer and analysed using FlowJo software (version 10, BD) and are expressed as mean (%) ± SEM. The dotted line represents the mean value for CD8^+^ T-cell responses to C1R + CBZ treatment. Statistical significance was determined using one-way ANOVA with Tukey’s MCT for *post hoc* analysis (^*^p < 0.05; ^**^p < 0.01; ^***^p < 0.001; ^****^p < 0.0001; Prism 10.0, GraphPad software). HLA class I genotyping is shown for each case (**Supplementary Table 1**).

**Figure 4:**
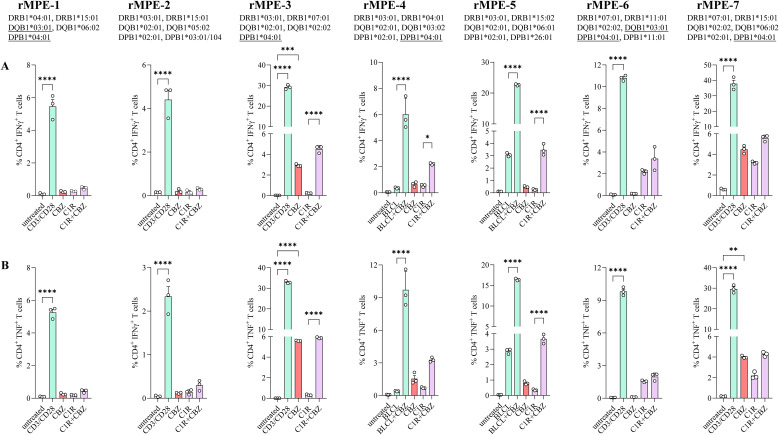
Drug-induced CD4^+^ T-cell recall responses in resolved MPE patients. PBMCs were treated *in vitro* with 25 μg/mL CBZ for 14 days. Drug-induced activation of CD4^+^ T-cells, via production of IFN-γ **(A)** and/or TNF **(B)**, was quantified following a 6-h restimulation with either no drug (untreated), CD3/CD28 beads, or autologous B-LCL + 25 μg/mL CBZ (positive controls), 25 μg/mL CBZ, or C1R parental cells in the absence or presence of 25 μg/mL CBZ. Triplicate data were acquired on a BD Fortessa X20 flow cytometer and analysed using FlowJo software (version 10, BD) and are expressed as mean (%) ± SEM. The dotted line represents the mean value for CD4^+^ T-cell responses to C1R + CBZ treatment. Statistical significance was determined using one-way ANOVA with Tukey’s MCT for *post hoc* analysis (^**^p < 0.01; ^***^p < 0.001; ^****^p < 0.0001; Prism 10.0, GraphPad software). HLA class II genotyping is shown for each case (**Supplementary Table 1**), with underlined allotypes shared with C1R parental cells.

Next, we explored whether convalescent MPE patients (range of 2 months to 10 years post-reaction), upon re-exposure to CBZ, display a CD8^+^ T-cell recall response and whether this was directed towards more than one HLA-I allotype expressed by the patient (rMPE-1–7; **Supplementary Table 1**). Day 14 CBZ-induced T-cell lines were restimulated with APCs (± 25 μg/mL CBZ) and then functionally assessed via Th1 cytokine production. Our data showed identical CD8^+^ T-cell responses toward CBZ (via both IFN-γ and TNF production) that were augmented by APCs expressing a range of HLA-I allotypes (**Figures 3A, B**). Self-presentation of CBZ to activate CD8^+^ T cells was also observed in four of seven patients (rMPE-3, rMPE-4, rMPE-5, rMPE-7) (**Figures 3A, B**). Stimulation in the presence of C1R expressing HLA-A/B matched to the patient produced greater cytokine production than parental C1R for at least one HLA-I in six of seven patients (excluding rMPE-5). For rMPE-5, we noted high levels of CD8^+^ T-cell activation towards the C1R parental line in the presence of a drug that was either equal to (**Figure 3A**) or greater than (**Figure 3B**) responses towards C1R.B*15:01 and C1R.B*58:01 cell lines. Both the C1R parental cell line and rMPE-5 express HLA-C*04:01, suggesting that CD8^+^ T-cell drug responsiveness is most likely triggered by this allomorph. Collectively, HLA coverage encompassed seven HLA-A allotypes (HLA-A*02:01, HLA-A*03:01, HLA-A*24:02, HLA-A*26:01, HLA-A*29:02, HLA-A*31:01, HLA-A*32:01), five HLA-B allotypes (HLA-B*07:02, HLA-B*18:01, HLA-B*35:01, HLA-B*40:02, HLA-B*44:03), and one HLA-C allotype (HLA-C*04:01). Similarly, CD4^+^ T-cell responses were examined in the rMPE cohort (**Figure 4**). Four cases showed reactivity to CBZ (rMPE-3 and rMPE-7, with IFN-γ in rMPE-7 trending toward significance, *p* = 0.1423) and/or to drug-pulsed APCs (rMPE-3, rMPE-4, and rMPE-5, with TNF in rMPE-4 trending toward significance, *p* = 0.1476). Collectively, our data suggest that convalescent MPE patients retain durable, polyclonal CD8^+^ T-cell recall responses to CBZ that are restricted by multiple HLA class I allotypes, including HLA-A, HLA-B, and HLA-C, with accompanying but variable CD4^+^ T-cell reactivity, highlighting long-term immunological memory despite clinical resolution.

### 3.3 CBZ-induced recall responses towards additional antiseizure medications

**Figure 5:**
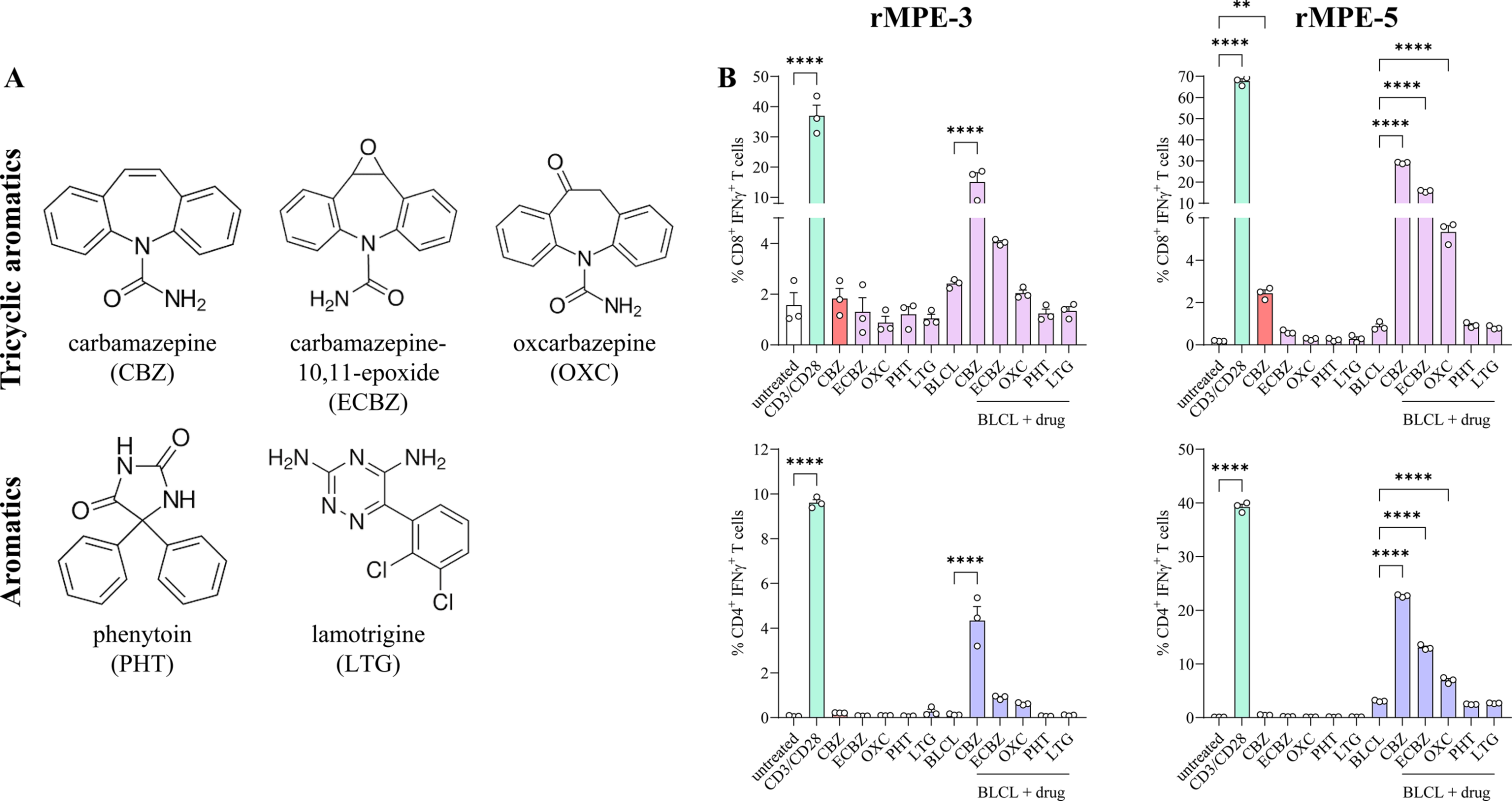
CBZ-specific T cells cross-react towards related antiseizure medications. **(A)** Commonly prescribed ASMs defined by chemical structure. **(B)** PBMCs derived from rMPE-3 and rMPE-5 were treated *in vitro* with different ASM drugs for 14 days. Activated CD8^+^ T-cell-producing IFN-γ were quantified following a 6-h restimulation with either no drug (untreated), CD3/CD28 beads (positive control; green), 25 μg/mL ASM drug alone, or autologous B-LCLs in the absence or presence of 25 μg/mL CBZ. Triplicate data were acquired on a BD Fortessa X20 flow cytometer and analysed using FlowJo software (version 10, BD). Statistical significance was determined using one-way ANOVA with Tukey’s MCT for post-hoc analysis (**p < 0.01, ****p < 0.0001; Prism 10.0, GraphPad software).

From the rMPE cohort, two cases (rMPE-3, rMPE-5; **Supplementary Table 1**) were selected based on sample availability to determine whether CBZ-induced memory T cells generated following primary CBZ exposure exhibited reactivity towards alternative antiseizure medications. Compounds included the CBZ metabolite carbamazepine-10,11-epoxide (ECBZ), the structural derivative oxcarbazepine (OXC), and alternative antiseizure drugs PHT and LTG (**Figure 5A**). Day 14 CBZ-induced T-cell lines were restimulated with 25 μg/mL drug alone or autologous B-LCLs (± 25 μg/mL drug), with T-cell recognition measured by IFN-γ production. For each case, the drug alone did not induce significant T-cell responses. However, drugs in the presence of autologous B-LCL showed reactivity towards the ECBZ metabolite and OXC structural derivative for both CD8^+^ and CD4^+^ T cells (trending in rMPE-3). No responses were detected for PHT or LTG in either case (**Figure 5B**). Of note, both patients had received CBZ alone at the time of reaction, without prior exposure to OXC, PHT, or LTG. Drug-tolerant controls were not included due to consistently poor expansion and weak functional responses following *in vitro* CBZ stimulation, consistent with our previous observations in CBZ-induced SJS/TEN cohorts (27).

### 3.4 Emergence of drug-induced TCR clonotypes following CBZ exposure

Next, we examined the CBZ-MPE TCR repertoires to determine whether there was clonotypic remodelling following drug exposure, as reported previously in SJS/TEN patients (27). Based on sample availability, CBZ-reactive T-cell lines were established from four rMPE cases (rMPE-3, rMPE-4, rMPE-5, rMPE-7; **Figure 3A**) and restimulated for 4 h with APCs either in the absence (untreated) or presence of CBZ (treated, 25 μg/mL). Given that polyclonal T-cell responses towards multiple HLA allotypes were observed, autologous B-LCLs in the presence of 25 μg/mL CBZ were used as stimulatory APCs to identify global CBZ-TCR clonotypes, except for rMPE-7 (stimulated with C1R.A*02:01 and C1R.A*03:01). T cells were segregated based on phenotype and activation status (non-activated: CD8^+^IFN-γ^−^ or activated: CD8^+^IFN-γ^+^) and then single-cell sorted for subsequent αβTCR repertoire analysis by multiplex PCR and Sanger sequencing. Complete TCR repertoire information is reported in **Supplementary Table 4**.

**Figure 6:**
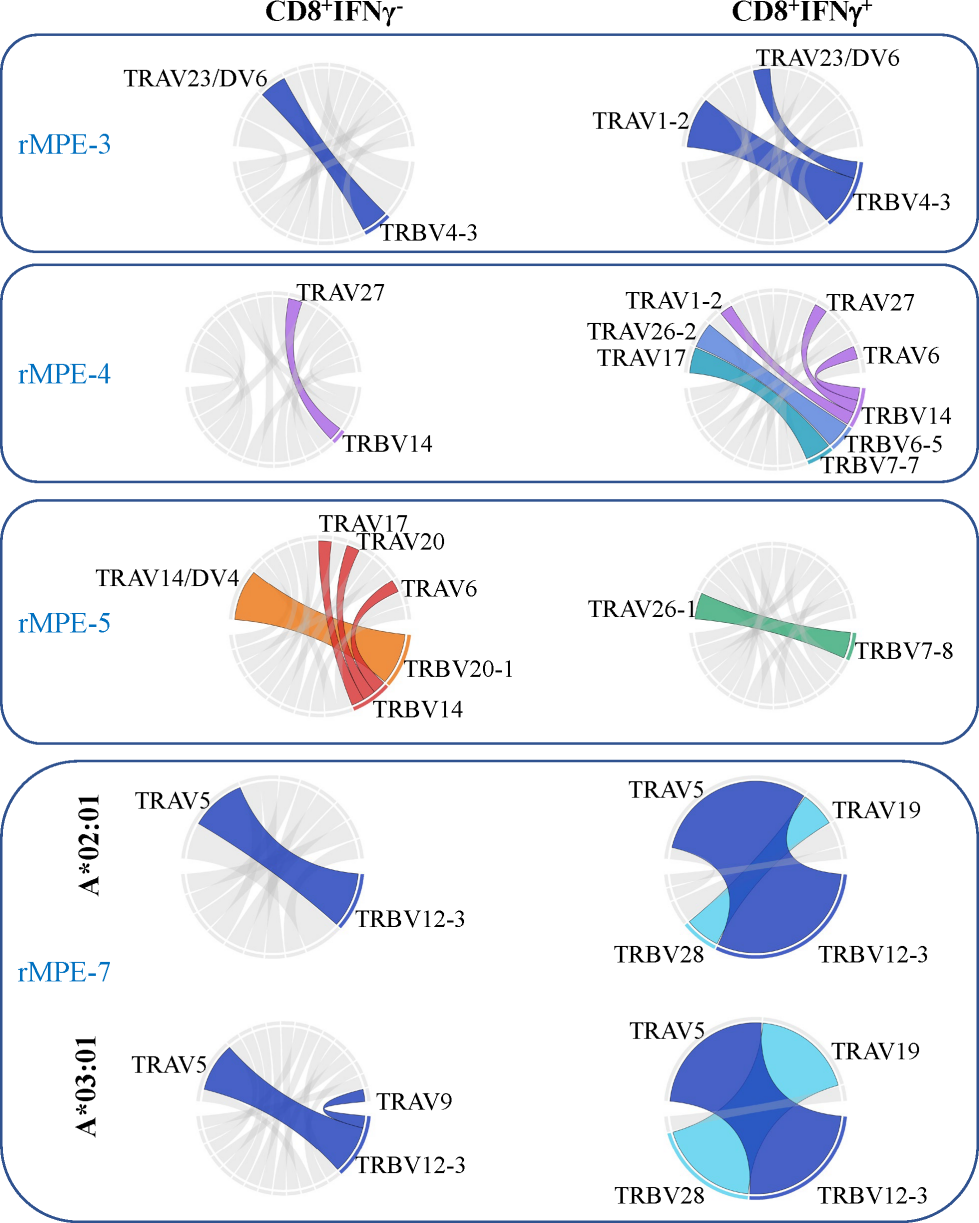
Resolved CBZ-MPE cases have a private TCR repertoire. **(A)** Chord plots for rMPE-3, rMPE-4, and rMPE-5 show private αβTCR clonotype pairings for both CD8^+^IFNγ^−^ (denoted as CD8) and CD8^+^IFNγ^+^ (denoted as IFN-γ) T-cell subsets. **(B)** Chord plots for rMPE-7 reveal a shared TRAV19_TRBV28 when day 14 CBZ-exposed T cells were restimulated with either C1R.A*02:01 or C1R.A*03:01 APCs in the presence of CBZ. TCR repertoire data were analysed using the web tool TCR_Explore (42), with major clonotypes shown in colors. **Supplementary Table 4** contains complete TCR sequences.

For resolved cases (rMPE-3, rMPE-4, and rMPE-5), sequencing of αβTCRs following restimulation of drug-specific T-cell lines with autologous B-LCLs in the presence of CBZ revealed polyclonal yet private CBZ-specific repertoires. Here, expansion of a single major clonotype was observed in both rMPE-3 (paired α_β variable genes; TRAV1-2_TRBV4-3) and rMPE-5 (TRAV26-1_TRBV7-8). While rMPE-4 displayed two major clonotypes, TRAV17_TRBV7–7 and TRAV26-2_TRBV6-5 (**Figure 6**). Given that we showed broad CD8^+^ T-cell responses towards different HLA allotypes in both active and resolved MPE patients (**Figures 1**, **3**), we wanted to investigate whether the same or different TCRs were responding to CBZ in the context of multiple HLA. Here, day 14 CBZ-exposed CD8^+^ T cells from rMPE-7 were separately restimulated with C1R.A*02:01 and C1R.A*03:01 prior to single-cell sorting of IFN-γ-producing T cells. TCR repertoire analysis identified a high-frequency TRAV5_TRBV12–3 clonotype in both activated and non-IFN-γ-activated CD8^+^ T cells, suggesting this clonotype may also be present within activated bystander T cells rather than representing exclusively drug-specific responses. In contrast, the TRAV19_TRBV28 clonotype emerged exclusively in CBZ-activated CD8^+^ T cells across these two stimulations (**Figure 6**). Finally, the four rMPE patients exhibited a private TCR repertoire, with no shared clonotypes being identified (**Supplementary Table 4**).

### 3.5 Transcriptomic analysis reveals molecular signatures of divergent CBZ-induced adverse reactions

This study cohort comprised individuals from mixed ethnicities, with CBZ-MPE cases prospectively recruited from Australia and CBZ-SJS cases retrospectively recruited from Hong Kong and Malaysia. Although same-region comparisons of mild and severe disease were not feasible due to the rarity of CBZ-SJS in Australia, potential ethnic bias was addressed by using a mixed-ethnicity tolerant control group (Australia and Hong Kong) as the baseline comparator for transcriptomic analyses (**Supplementary Table 1**). Although cohort HLA heterogeneity may contribute to inter-individual variability, transcriptional signatures are more strongly influenced by cellular composition, activation state, and functional immune responses rather than ancestry alone (58).

**Figure 7:**
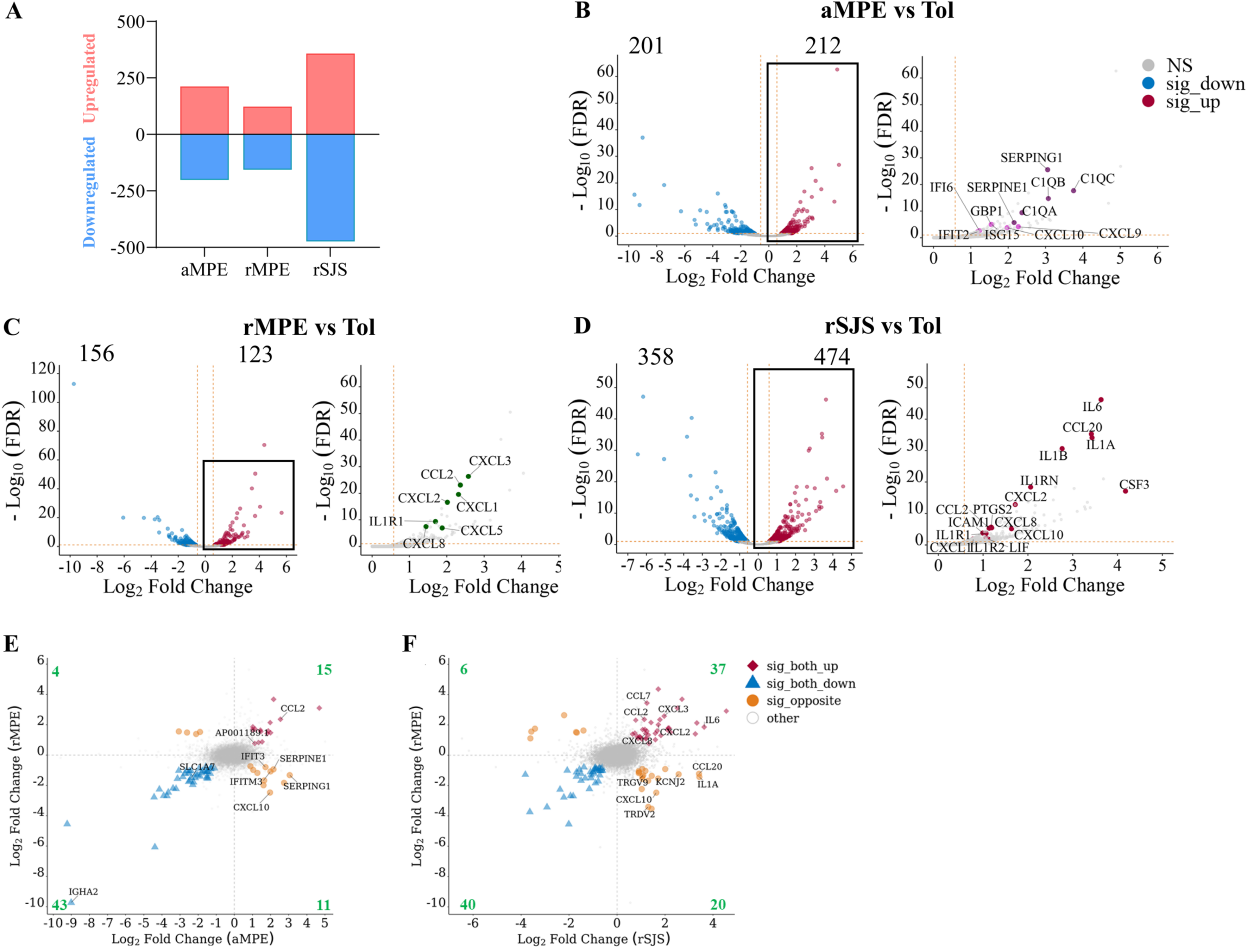
Differential transcriptional signatures and pathways of CBZ-induced rash state and severity. **(A)** Total number of upregulated and downregulated DEG transcripts identified for each group compared to Tol cases. **(B–D)** Volcano plots (49) of all data (left) and the zoomed-in section (indicated by the black box), highlighting specific genes (right) from key enriched pathways based on the g:Profiler pathway analysis (**Supplementary Table 5S4–S6**). The *x*-axis represents the log_2_FC, and the *y*-axis represents the –log_10_ of FDR. **(B)** aMPE vs. Tol: IFNII pathway (pink) and complement cascade (purple). **(C)** rMPE vs. Tol: highlighting the neutrophilic pathway (green). **(D)** rSJS vs. Tol: highlighting the genes from IL-10 (red) signaling. **(E, F)** Total numbers of significant DEGs (green numbers), significantly upregulated in both (red diamonds), significantly downregulated in both (blue triangles), or significantly opposite (orange circles). Grey open circles represent genes that are significant in only one analysis or nonsignificant in both analyses. A threshold of log_2_FC > 0.58 and FDR < 0.1 was applied. **(E)** Correlation plot comparing log_2_FC of aMPE vs. Tol (*x*-axis) with rMPE vs. Tol (*y*-axis) (**Supplementary Table 5S7**). **(F)** Plot comparing log_2_FC of rSJS vs.Tol (*x*-axis) with rMPE vs. Tol (*y*-axis) (**Supplementary Table 5S8**).

To delineate genetic signatures underlying CBZ-induced mild versus severe cADRs, whole-transcriptome RNA-Seq was performed on PBMCs obtained from aMPE (< 9 days post-reaction), rMPE (2–123 months convalescence), rSJS (3–246 months post-reaction), and drug-Tol cases (**Supplementary Table 1**). A total of 16,948 transcripts were analysed, with variance across groups assessed by PCA. Tol cases overlapped with both mild and severe reactions (**Supplementary Figure 4A**), indicating that tolerance- and disease-associated features did not dominate the principal variance axes. Notably, a clear separation was observed between aMPE and rSJS along the PC1 axis. Relative to Tol cases, transcriptional dysregulation was more pronounced in rSJS than in aMPE or rMPE (**Figure 7A**; **Supplementary Table 5S1–S3**). Consistent with the PCA, hierarchical clustering of significantly differentially expressed genes (FDR < 0.1; logFC ± 1) across comparisons (aMPE vs. Tol, rMPE vs. Tol, and rSJS vs. Tol) further demonstrated robust separation between aMPE and rSJS (**Supplementary Figure 4B**).

Drug-induced MPE is generally considered a milder nonbullous form of delayed (type VI) cADR, with the precise immunological subtype remaining incompletely defined. It occurs within a broader spectrum of cADRs that includes severe immunopathologies such as DRESS and SJS (1). To explore this spectrum, we assessed PBMC transcriptional correlations across disease states relative to drug-Tol cases. Tol cases served as a baseline reference, enabling comparison of transcriptional changes in relation to reaction severity (**Figure 7**). Correlation analysis showed that > 88% of DEGs were distinct between rMPE and rSJS, underscoring their divergent immunopathology, with rSJS driven largely by cytotoxic and cytokine-producing CD8^+^ T cells. MPE lacked DRESS-like features such as eosinophil recruitment or Th2 cytokines (IL-4, IL-5, IL-13). In aMPE, upregulated transcripts were enriched for interferon signalling (*GBP1*, *CXCL9*, *CXCL10*, *IFITM1*, *IFITM3*, *IFIT2*, *IFIT3*, *ISG15*, *IFI6*) and complement activation (*C1QA*, *C1QB*, *C1QC*, *SERPING1*, *SERPINE1*), reflecting strong innate immune activity (**Figure 7B**; **Supplementary Table 5S4**). Downregulated transcripts involved antigen processing, peptide binding, and MHC pathways. Together, these findings suggest complement-driven innate responses dominate acute MPE pathogenesis rather than classical type IVb/c hypersensitivity (8). By contrast, rMPE showed persistent upregulation of chemokine, cytokine, TNF, and NF-κB pathways, including IL-10 (*CCL2*, *CXCL1*, *CXCL2*, *IL6*, *IL1R1*, *CXCL8*), IL-17 (*CCL7*, *CXCL3*, *CCL2*, *CXCL1*, *CXCL2*, *IL6*, *CXCL8*, *CXCL5*), and IL-18 (*CXCL3*, *CCL2*, *CXCL2*, *IL6*, *SPP1*, *CXCL*) signalling (**Figure 7C**; **Supplementary Table 5S5**). Overall, only 11.8% (73/619) of DEGs overlapped between aMPE and rMPE (**Figure 7E**). Here, 11 DEGs showed opposite regulation, being upregulated in aMPE but not rMPE; these included IFN-inducible genes that regulate cellular processes (*IFIT3*, *IFITM3*), a proinflammatory cytokine (*CXCL10*), and complement components (*SERPINE1*, *SERPING1*), consistent with heightened inflammatory cell recruitment during acute disease (**Figure 7E**, bottom right; **Supplementary Table 5** **[S7]**). However, we observed that 43 shared DEGs were downregulated, including 15 immunoglobulin transcripts, most notably *IGHA2*, suggesting loss of IgA^+^ B cells (**Figure 7E**, bottom left; **Supplementary Table 5S1, S2**).

We observed that rSJS demonstrated similar inflammatory pathways to rMPE, but with higher significance for IL-10 and IL-17 signalling (adjusted *p* = 8.96 × 10^−19^ and 2.53 × 10−13^-^¹³, respectively). These cases were distinguished by cytotoxic leukocyte pathways (*ICAM1*, *GZMB*, *RNF19B*, *SERPINB9*, *SLAMF6*, *LAG3*, *KLRC1*, *PRF1*, *CADM1*, *IL18RAP*, *KLRD1*, *SLAMF7*) and acute inflammatory responses (*IL6*, *IL1A*, *IL1B*, *IL1RN*, *F3*, *FFAR2*, *PTGS2*) (**Figure 7D**; **Supplementary Table 5S6**). Overall, DEG overlap between rMPE and rSJS was modest at 10.2% (103/1,008; **Figure 7F**). Despite sampling many years post-onset (**Supplementary Table 1**), rSJS retained a strong inflammatory profile, with upregulated cytokine/chemokine transcripts (*CCL20*, *CXCL10*, *IL-1A*, *KCNJ2*, *FOSB*; **Figure 7F**, bottom right). rSJS also uniquely upregulated genes implicating a Vγ9Vδ2 T-cell subset (*TRGC1*, *TRGV9*, *TRDC*, *TRDV2*). Despite being on opposite ends of the disease spectrum, both rMPE and rSJS shared upregulation of cytokines (*IL-1R1*, *IL-6*) and chemokines (*CXCL1*, *CXCL2*, *CXCL3*, *CXCL8*, *CCL2*, *CCL7*; **Figure 7F**, top right; **Supplementary Table 5S8**). As noted above, whilst there were many genes and pathways (including IL-10, IL-17, and IL-18) commonly upregulated in both resolved mild and severe disease settings, there were statistical differences associated with their magnitude relative to disease state. For example, *IL-1B*, *IL-1RN*, and PTGS2 were more highly expressed in rSJS, while *CCL7* and *CCL2* predominated in rMPE (**Supplementary Figure 5**). These results suggest that rSJS remains perpetually primed through proinflammatory and cytotoxic programs, potentially heightening susceptibility to future severe drug reactions and contributing to long-term complications.

Lastly, we examined whether previously reported genome-wide association studies (GWAS) risk markers linked to ASM-induced MPE or SJS, including *DOCK10*, *ABCA1*, *CD24*, *HLA­E*, *CD9*, and *CFHR4* (59, 60), were represented in the MPE dataset. Of these, only *CD24* was significantly dysregulated in rMPE vs. Tol (FDR: 0.0096, logFC: − 1.17) and rSJS vs. Tol (FDR: 0.036, logFC: 0.90), but not aMPE vs. Tol. Other genes, *DOCK10* and *ABCA1*, were detected but not significantly altered, while *HLA-E* and *CFHR4* were below the threshold of detection (**Supplementary Table 5S7, S8**). Overall, integrated PBMC transcriptomic pathways identified distinct immune features across CBZ-induced disease states, summarised in **Table 1**.

## 4 Discussion

Causative medications that trigger DHRs often begin as mild symptoms but may rapidly progress to severe pathology if the drug is not promptly withdrawn (61). These reactions are classified by the immune cell types and inflammatory mediators involved (8). MPE is the most common and typically mild presentation, usually occurring 5–14 days after drug initiation but occasionally arising weeks to months later (62). MPE is idiosyncratic and primarily mediated by type IVb and IVc T-cell responses (61, 63), with broad HLA associations across both HLA-A (21–23, 25, 64) and HLA-B (20, 24, 25) allotypes. In contrast, SJS/TEN is a severe, type IVc reaction (8) with strong HLA associations that vary by culprit drug (65). While these immunological classifications remain useful, advances in multiomics, particularly combined TCR-seq and RNA-seq, now allow deeper insights into immune cell involvement and recognition mechanisms.

In this study, we investigated the effects of CBZ-induced MPE during active disease (< 9 days post-diagnosis) and after convalescence (> 2 months post-reaction) in the peripheral blood of patients. We focused on direct CD8^+^ T-cell activation in acute disease, the persistence of memory CD8^+^ T-cells bearing HLA-restricted clonotypes, and T-cell recognition of structurally related ASMs. In parallel, bulk transcriptomics was performed to distinguish MPE from severe cADRs such as SJS (66) and to define gene signatures differentiating active and resolved MPE.

We observed robust effector CD8^+^ T-cell responses, with IFN-γ and/or TNF production, frequently directed toward multiple HLA allotypes. These findings support cytotoxic T-cell involvement in both active (primary) and resolved (memory) disease and highlight HLA promiscuity in milder CBZ reactions, which is uncommon in severe cADRs (27, 65). Proinflammatory cytotoxic CD8^+^ T-cell responses were detected across APCs expressing several responder-matched HLA-A (A*02:01, A*03:01, A*24:02, A*26:01, A*29:02, A*31:01, A*32:01, A*33:03), HLA-B (B*07:02, B*15:01, B*18:01, B*35:01, B*40:02, B*44:03, B*49:01, B*51:01), and HLA-C*04:01 allotypes, with several previously reported as CBZ-MPE risk alleles (14, 23, 25, 67). Interestingly, in aMPE cases, CD4^+^ T-cell involvement in peripheral blood was limited, potentially reflecting migration to the skin, the primary site of pathology, whereas CD8^+^ T-cells remained detectable in circulation. In contrast, rMPE cases exhibited CBZ-reactive CD4^+^ and CD8^+^ memory T-cells in the periphery, indicating persistence of drug-specific immune memory following clinical resolution.

Interpretation of HLA findings should be considered in the context of the ethnically diverse composition of our cohort and the known population-specific nature of CBZ hypersensitivity associations. Unlike severe CBZ-induced SCAR phenotypes, such as SJS/TEN, which demonstrate strong enrichment for defined HLA risk alleles in specific ancestral groups, MPE susceptibility appears more genetically heterogeneous and is not consistently linked to a single HLA allele across populations (22, 23, 59). Consistent with this, we did not observe clear enrichment of a shared HLA allele in the MPE cohort compared to CBZ-tolerant controls, with several HLA class I alleles represented across both groups. As this study was not designed or powered as a genetic association study, larger ancestry-stratified cohorts will be required to more definitively assess HLA-association risk in CBZ-induced MPE.

Patients with CBZ-induced DHR are at increased risk of the same or more severe reactions when exposed to alternative ASMs. A study by Wang et al. (68) reported that 66.7% of CBZ-exposed patients experienced adverse reactions to the related drug OXC, driven by cross-reactive T-cell recognition of its tricyclic aromatic core. In SJS/TEN cases, CBZ-reactive T-cells cross-reacted with ECBZ and OXC, but not with ASMs containing only one or two aromatic rings (9, 27). Here, we assessed the drug reactivity of CBZ-reactive CD8^+^ T-cells from rMPE patients. Significant responses were observed to the CBZ metabolite ECBZ, trending responses to OXC, and no responses to PHT or LTG. While this suggests PHT and LTG may be suitable alternatives, individual evaluation remains essential, as secondary drug reactivity has been reported for PHT (30%–47.6%) and LTG (13%–29.4%) in CBZ-cADRs (68–71).

Next, we analysed the TCR repertoire of circulating CD8^+^ T-cells from rMPE patients to assess clonality and the presence of private versus public TCRs. Day 14 bulk T-cells were restimulated for 4 h with HLA-matched APCs (selected based on **Figures 1**, **2**) in the presence of CBZ, and activated CD8^+^ T-cells (IFN-γ^+^) were single-cell sorted for paired TCRα/β sequencing. Private CBZ-reactive TCR clonotypes were identified in rMPE-3 (TRAV1-2_TRBV4-3), rMPE-4 (TRAV17_TRBV7-7, TRAV26-2_TRBV6-5), rMPE-5 (TRAV26-1_TRBV7-8), and rMPE-7 (TRAV19_TRBV28). These private TCRs mirror patterns observed in CBZ-SJS/TEN cases (27), although those severe reactions were exclusively restricted to HLA-B*15:02.

Transcriptomic profiling of drug-induced mild or resolved disease remains limited. Bellon et al. used array-based analysis to show that non-CBZ MPE cases could cluster with either DRESS or SJS/TEN based on gene signatures (29), consistent with MPE encompassing both type IVb and IVc reactions (29, 72). In contrast, severe cADRs such as SJS/TEN are restricted to type IVc responses, with transcriptomics highlighting cytotoxic mediators including granulysin, perforin, and granzyme B (11, 66). Here, we compared transcriptional profiles across CBZ-induced disease states (aMPE vs rMPE) and across disease severity (rMPE vs. rSJS).

In blood, our data revealed limited transcriptional overlap (< 12%) between disease states and severities when using CBZ-tolerant cases as a baseline. For disease severity, overlapping upregulated transcripts were enriched in T-cell-related pathways, including IL-10, IL-17, and IL-18. However, severe disease (rSJS) exhibited greater dysregulation, with higher expression of proinflammatory cytokines IL-6, IL-1B, IL-1A, and PTGS2. In contrast, rMPE upregulated cytokines such as CCL7 and CCL2, monocyte chemoattractants linked to type IVa pathology (8, 73, 74). Active versus resolved MPE showed minimal transcript concordance, with aMPE biased toward innate pathways, including early complement activation, with all C1q complex genes detected. These findings align with a WGS study of phenytoin-induced MPE implicating complement factor H R4 (CFHR4) (59), although CFHR4 was not dysregulated in our dataset. Overall, distinct transcriptional signatures separate mild and severe CBZ-induced disease, involving both innate immune cells and T-cells.

Beyond T-cell signalling and inflammatory pathways, a notable feature of MPE was downregulation of IGHA2, the constant region of the B-cell receptor for IgA. Loss of IGHA2 in both active and resolved MPE suggests selective IgA deficiency, which is common in epilepsy (75) and has been linked to ASM use, with CBZ exerting the strongest effect on IgA^+^ B cells (76). This immunodeficiency is also more prevalent in atopic conditions, including allergies, asthma, and dermatitis (77–79). Further studies assessing serum IgA in MPE cases are needed to confirm this observation, which could not be evaluated here due to the lack of available patient serum. Additionally, we observed downregulation of the innate-like Vγ9Vδ2^+^ T-cell population (80), which has been reported to mount allergen-specific, Th1-polarised responses (81). While αβ T cells are the primary mediators of DHRs, our findings suggest that B cells and γδ T cells may also contribute to type IV–subtype specificity.

Historically, immune mechanisms underlying MPE have often been classified within the framework of type IV hypersensitivity subclasses, including DRESS-associated Th2/eosinophilic responses and SJS/TEN-associated T-cell-mediated responses (8). However, our transcriptomic analyses did not identify evidence of dominant Th2-associated signalling (IL-4, IL-5, IL-13) or eosinophilic pathways in MPE. Instead, we observed a broader range of immune features, including monocyte-associated inflammatory pathways, cytotoxic CD8^+^ T-cell signatures, and neutrophil-associated chemokine expression (**Table 1**).

## 5 Limitations of the study

First, our analyses were performed exclusively using peripheral blood-derived T cells, without matched skin tissue. Recent studies (30, 31) have demonstrated that MPE is predominantly a skin-localised immune process driven by tissue-resident T-cell responses, with comparatively limited systemic clonal expansion relative to SJS/TEN. However, peripheral blood remains a clinically accessible compartment for investigating antigen-specific immune activation, immune memory, and circulating CBZ-reactive T-cell responses. Accordingly, this study was designed to complement tissue-focused investigations by characterising the systemic and memory immune features associated with CBZ-induced MPE.

Second, because circulating CBZ-reactive T cells are typically present at low frequency, particularly in milder cutaneous reactions such as MPE (14, 27, 82, 83), *in vitro* expansion was used to generate sufficient drug-responsive cells for functional and TCR repertoire analyses. This approach enabled detailed interrogation of otherwise difficult-to-detect drug-responsive populations.

Third, the cohort size was modest and included ethnically diverse individuals with heterogeneous HLA backgrounds, which may reduce the ability to detect shared HLA associations or conserved public TCR signatures. Consistent with the complex and multifactorial nature of genetic susceptibility in MPE (22, 59), several HLA alleles identified in CBZ-MPE cases were also present in tolerant controls. Additional variability may reflect differences in clinical phenotype, timing of sample collection, and treatment exposure. Furthermore, active and resolved cohorts were independently recruited rather than longitudinally sampled, limiting direct assessment of immune mapping following clinical recovery.

Finally, drug reactivity analyses were performed in a limited number of patients and without sufficient drug-tolerant comparator controls. The lack of such controls reflects the challenges of generating sufficient functional responses from drug-tolerant patients *in vitro*. Future studies incorporating paired blood–skin sampling, longitudinal analyses, and larger multi-ethnic cohorts will be important to further refine our understanding of tissue-specific and circulating immune mechanisms underlying CBZ-induced MPE.

In conclusion, this study characterised circulating T-cell responses and transcriptional signatures in CBZ-induced MPE. We observed both acute and long-lived CBZ-specific CD8^+^ T-cell responses, with drug reactivity to structurally related tricyclic compounds. TCR sequence analysis revealed a private repertoire, with a single dominant clonotype in each patient. Transcriptomic profiling identified diverse immune and inflammatory gene signatures that extend beyond traditional type IV hypersensitivity classifications, highlighting the immunological complexity of CBZ-induced MPE and supporting more individualised approaches to diagnosis and disease stratification.

## Data Availability

The original contributions presented in the study are included in the article/**Supplementary Material**. Further inquiries can be directed to the corresponding authors.
